# Supplementary material for: Supraglottic airway devices for blind endotracheal intubation: A systematic review
Source: PLoS One. 2024 Dec 11;19(12):e0315301. doi: 10.1371/journal.pone.0315301 (PMC11634001; doi:10.1371/journal.pone.0315301)
Supplement: S2 Table — (DOCX) [file pone.0315301.s004.docx]

**S2 Table. Risk of bias in the included studies.**

| **Author, year (country)** | **Random sequence generation** | **Allocation Concealment** | **Blinding of outcome assessors** | **Incomplete outcome data** | **Selective reporting of results** |
| --- | --- | --- | --- | --- | --- |
| **Zuercher, 2022, Switzerland** | Low risk | Low risk | High risk | Low risk | Low risk |
|  |  |  |  |  |  |
| **De Rosa, 2018, Italy** | High risk | High risk | High risk | Low risk | Low risk |
|  |  |  |  |  |  |
| **López Correa, 2016, Spain** | Some concerns | Low risk | High risk | Low risk | Low risk |
|  |  |  |  |  |  |
| **Ott, 2020, Germany** | Low risk | High risk | Some concerns | Low risk | Low risk |
|  |  |  |  |  |  |
|  |  |  |  |  |  |
|  |  |  |  |  |  |
| **Fun, 2007, Singapore** | Low risk | Low risk | Some concerns | Low risk | Low risk |
|  |  |  |  |  |  |
| **Gawlowsky, 2017, Polonia** | Low risk | Some concerns | High risk | Low risk | Low risk |
|  |  |  |  |  |  |
|  |  |  |  |  |  |
|  |  |  |  |  |  |
| **Melissopoulou, 2014, Grecia** | Low risk | Low risk | High risk | Low risk | Low risk |
|  |  |  |  |  |  |
| **Kurowski, 2015, Poland** | Low risk | High risk | Some concerns | Low risk | Low risk |
|  |  |  |  |  |  |
|  |  |  |  |  |  |
| **Uribe-Valencia, 2014, Colombia** | High risk | High risk | Some concerns | Low risk | Low risk |
|  |  |  |  |  |  |
| **Shavit, 2015, Israel** | Low risk | High risk | Some concerns | Low risk | Low risk |
|  |  |  |  |  |  |
|  |  |  |  |  |  |
| **Ott, 2015, Germany** | Low risk | High risk | High risk | Low risk | Low risk |
| **Komasawa, 2014, Japan** | Low risk | High risk | Some concerns | Low risk | Low risk |
|  |  |  |  |  |  |
| **Michalek, 2010, United Kingdom** | Low risk | High risk | Some concerns | Low risk | Low risk |
|  |  |  |  |  |  |
| **Alonso, 2012, Spain** | High risk | High risk | Low risk | Low risk | Low risk |
|  |  |  |  |  |  |
| **Bielski, 2018, Poland** | Low risk | Some concerns | High risk | Low risk | Low risk |
|  |  |  |  |  |  |
|  |  |  |  |  |  |
| **Szarpak, 2015, Poland** | Low risk | Some concerns | High risk | Low risk | Low risk |
|  |  |  |  |  |  |
| **Anuradha, 2017, India** | Low risk | Low risk | High risk | Some concerns | High risk |
|  |  |  |  |  |  |
| **Darlong, 2011, India** | Low risk | Low risk | Some concerns | Low risk | Low risk |
|  |  |  |  |  |  |
| **Halwagi, 2012, Canadá** | Low risk | Low risk | High risk | Low risk | High risk |
|  |  |  |  |  |  |
|  |  |  |  |  |  |
| **Kapoor, 2014, India** | Low risk | Low risk | High risk | Low risk | Low risk |
|  |  |  |  |  |  |
| **Karim, 2011, USA** | Low risk | Low risk | High risk | Low risk | Low risk |
|  |  |  |  |  |  |
| **Liu, 2008, Singapore** | Low risk | Low risk | High risk | Low risk | Low risk |
|  |  |  |  |  |  |
|  |  |  |  |  |  |
| **Malhotra, 2016, India** | Low risk | Low risk | Low risk | Low risk | Low risk |
|  |  |  |  |  |  |
|  |  |  |  |  |  |
| **Neoh, 2012, Malaysia** | Low risk | Low risk | Low risk | Low risk | Low risk |
|  |  |  |  |  |  |
|  |  |  |  |  |  |
| **Teoh, 2007, Singapore** | Low risk | Low risk | High risk | Low risk | Low risk |
|  |  |  |  |  |  |
| **Sethi, 2017, India** | Low risk | Low risk | High risk | Low risk | Low risk |
|  |  |  |  |  |  |
|  |  |  |  |  |  |
|  |  |  |  |  |  |
| **Garzón, 2014, Spain** | Low risk | Low risk | Some concerns | Low risk | Low risk |
|  |  |  |  |  |  |

High risk indicates high risk of bias; low risk, low risk of bias; some concerns risk, some concerns risk of bias because of lack of detailed reports.
