# Supplementary material for: Supraglottic airway devices for blind endotracheal intubation: A systematic review
Source: PLoS One. 2024 Dec 11;19(12):e0315301. doi: 10.1371/journal.pone.0315301 (PMC11634001; doi:10.1371/journal.pone.0315301)
Supplement: Attachment — Submitted filename: Rebuttal letter.docx [file pone.0315301.s005.docx]

Neiva July 18, 2024

Dra.

**Emily Chenette**

Editor-in-Chief

Journal PLOS ONE

Dear Dra Emily,

Thank you very much for reviewing our manuscript titled **“Supraglottic airway devices for blind endotracheal intubation: A systematic review”** in PLOS ONE. We appreciate the comments made by each of the reviewers and we have responded point by point to each of the questions raised by them. Additionally, in this new version we have updated the search period. In our opinion, the quality of the manuscript has increased.

We have attached a tracked version of the manuscript so you can see how and where we have modified the original manuscript.

Looking forward to hearing from you,

Sincerely,

**Jorman Harvey Tejada Perdomo**

Corresponding Author

**Reviewer #1: Comments**

*Introduction*

- Comment #1: You quote a failure rate of DL at 30%, but the source cited is a study on prehospital intubations by EMTs. Surely this high failure rate does not apply to the general perioperative setting under elective circumstances – please clarify what you mean in the text so as not to confuse the reader into thinking that we only successfully intubate 70% of patients using DL in the OR.

*Thanks for your comment. We have clarified the information in the manuscript.*

- Comment #2: The ASA Emergency Airway algorithm suggests attempting a SGA if mask ventilation isn’t adequate after a failed intubation, not blind intubation through an SGA. If an emergency airway is required in a ‘cannot intubate, cannot ventilate’ scenario, their algorithm suggests considering alternative intubation while preparing for an emergency airway, under which a blind intubation through an SGA might.

*Thanks for your comment. We have introduced the suggested changes in the manuscript.*

*Methods*

- Comment #1: I can’t find an reasoning for which clinical trials were included – were all types of trials on blind intubation using SGAs included in the review and attempted meta-analysis? Was the meta-analysis performed including human patients, mannikins and cadavers?

*Thanks for your comment. Following your suggestions we agree to present the study data as a systematic review.*

- Comment #2: In the results section you mention that trials using one intubation device only were excluded, but it is still unclear what the methodology were of the trials included. Are these RCTs, and were they comparing devices or a device vs control? How did the study design differ between the different study populations?

*Following your comment, we have clarified the inclusion criteria in this new version.*

- Comment #3: Synthesis of information: Please expand on what was done in terms of the qualitative analysis briefly mentioned prior to the statistical analysis, and please comment on how it affected your selection of studies to include.

*Thanks for your comment. We have expanded the Synthesis of information in the document.*

*Statistical analysis*

- Comment #1: Please review the section on statistical analysis and considering clarifying your methodology regarding the meta-analysis performed. How did the quality of the studies on humans vs cadaver compare? It seems to me a stretch to include both in a pooled data set, as they can have very different circumstances and different sets of bias.

*Following the reviewer’s suggestion, we have reformatted the study data as a systematic review. Consequently, we have removed the statistical analysis section and clarified the methodology throughout the manuscript.*

- Comment #2: Studies using different types of statistical analysis were included in the meta-analysis which raises some concern over the quality of the analysis performed. The mean is useful in normally distributed populations but not those with skewed data, for which median is a more accurate tool; while it is certainly possible to convert median to mean using the data sets provided, it makes me concerned that very different populations were included in the meta-analysis if you had to convert reported medians to means, undermining the findings.

*We appreciate your evaluation. We have reformatted the study data as a systematic review. Consequently, we have removed the statistical analysis section and clarified the methodology throughout the manuscript.*

- Comment #3: This is an interesting study on a highly relevant topic – it is pertinent to be able to establish an airway quickly in patients presenting with a ‘cannot intubate, cannot ventilate’ scenario. For this purpose, intubating SGAs can be invaluable in the emergent setting and assessing their utility in terms of success rates and ease of intubation measured by intubating time is of great interest not only to anesthesiologists but to any clinician managing emergent situations. I commend the authors for their strong efforts put into this study. However, my overall recommendation is to reject the manuscript in it’s current form as I am concerned that the data presented here is too variable to be included in a meta-analysis. The clinical trials included in the study would all suffice for a systematic review, however when presented as a meta-analysis, the reader is led to believe that the results of studies on humans, mannikins and cadavers alike are all comparable when in fact these studies vary greatly in scope and application. If the authors can consider revising their manuscript and present their data as a systematic review alone, this data would be a valuable contribution to the discussion on the use of SGA for emergent airway management in the adult population.

*Thank you for your comments. We follow your recommendations, and we agree to present the work as a systematic review alone. These data are a valuable contribution to the discussion regarding the use of SGDs for emergent airway management in the adult population*

**Reviewer #2: Comments**

- Comment #1: I appreciate the effort, and time spent for the work. The topic chosen is crucial and has strong practical application. Overall the methodology employed and reported is good and standard, and manuscript is written well. However, I find a major issue with the total number of citations(articles) found as search results.

*Thanks for your precision. We have clarified the information suggested in the manuscript.*

- Comment #2: There is discrepancies between the abstract, methodology description and results and PRISMA flow diagram. Please have a look.

*Thanks for your comment. We have introduced the suggested changes in the manuscript.*

- Comment #3: Other minor aspect that need to attentions are- PICOT/PICOS can be clearly mentioned as a table.

*Thanks for your valued comment. We have clarified the information suggested in the manuscript. The studies were synthesized using a well-formulated Population, Intervention, Control and Outcomes (PICO) study design and listed in a table.*

- Comment #4: Timeframe for each database can be mentioned.

*Thanks for your comment. We have introduced the suggested changes in the manuscript.*

- Comment #5: Grey literature appears to be excluded that needs to be mentioned. The authors can also comment on the TSA/power.

*Thanks for your comment. We took your suggestion into account and expanded the information in the document.*
