# Supplementary material for: Supraglottic airway devices for blind endotracheal intubation: A systematic review
Source: PLoS One. 2024 Dec 11;19(12):e0315301. doi: 10.1371/journal.pone.0315301 (PMC11634001; doi:10.1371/journal.pone.0315301)
Supplement: Attachment — Submitted filename: Review.docx [file pone.0315301.s006.docx]

Thank you for the opportunity to review the manuscript “Supraglottic airway devices for blind endotracheal intubation: A systematic review.” by Tejada-Perdomo et al.

The authors have made a great effort to synthesize the available literature on this topic. I have some points that need to be addressed and would be happy to see the revised manuscript after a major revision.

Methods:

- Have you registered this SR at Prospero? Please state the PROSPERO registration ID in the method section.
- The search terms used are focused on prehospital emergency medicine. However, in the eligibility criteria, it is stated that “The studies involved patients under general anesthesia for surgery, simulation manikins, or cadavers.” Were no studies found in the prehospital setting? This would result from the search and not an inclusion or exclusion criteria. In-/Exclusion criteria should be defined upfront and, in this case include the prehospital setting. Please revise this issue; if no prehospital studies were found, briefly state this in the results.
- Please indicate if a professional librarian performed the search and defined the search strategy.
- Exclusion of pediatric studies: What is the reason for excluding these studies? Airway management is even more time-sensitive in children compared to adults. Further, hypoxia is the leading reversible/preventable cause of cardiac arrest. Since you have identified pediatric studies, I recommend including those in your systematic review and reporting them as a subgroup. This would enhance the quality of your work.
- Contact of the study authors: This is valuable information and a great effort from the team to (try to) contact the study team. How many responses did you get? If none, you can also state this.

Results:

- Please comment on how many studies were prospective, retrospective, or randomized controlled trials. This information can also be included in Table 2 or 3. Another reviewer previously mentioned this.
- It is crucial that you stated who performed the SGD intubation. By “anesthesiologists,” do you mean that the studies were conducted in the OR? For example, Anesthesiologists work as prehospital physicians in Germany due to the lack of a dedicated emergency medicine specialty. I recommend reporting the specialty (anesthesiology, internal medicine, emergency medicine,…) as stated in the study or the level of training (resident, consultant).
- You have included studies in humans, mannequins, and cadavers. Please report if the success rates were on humans, mannequins or cadavers.
- “Among the studies involving patients, LMA Fastrach and i-gel demonstrated the shortest intubation times (11,19,24,25,27,28,36,37,40,41).” Please state the intubation times.
- You have extracted data on comparing SGA vs. direct or video laryngoscopy. Can you please elaborate on this? This is the proper comparator for a failed airway.

Discussion:

- Differences in intubation time: Please note that the differences can be due to the definitions used for “intubation time.” Were you able to extract the definition from the respective studies? There is a big difference when the intubation time is measured from positioning the ET tube through an already placed LMA compared to when the LMA has to be placed and removed afterward. This should be a part of your discussion and can explain fast intubation times (4 seconds!). Reference 40: *“Times for tracheal intubation were measured from the beginning of first insertion of the laryngeal mask conduit to completion of tracheal intubation. This timing included the time required for removal of the laryngeal mask conduit, confirmation of ventilation with capnography, and use of the Macintosh laryngoscope where intubation with the LMA Fastrach ™ or LMA CTrach ™ systems had failed.”*

Reference 28: “*Insertion time (s) (defined as the time taken from the moment the ETT was inserted into the SGA device till an adequate EtCO_2_ trace was obtained).”*

If this is not accounted for in your discussion, the blind intubation time should be interpreted cautiously. If there is too much heterogeneity in the definitions used, this should be stated as well.

- “The LMA Fastrach and i-gel exhibited the highest first-attempt success rates, followed by iLTS-D2 and S.A.L.T., all surpassing an 80% success rate. These results are satisfactory and promising from a clinical perspective.” A rate of 80% is not satisfactory. Current resuscitation guidelines state that an expert needs a 95% success rate within two attempts. This should be the standard against which the SGAs are compared. Please revise your discussion accordingly.
- “While it is crucial for an anesthesiologist to establish a definitive airway rapidly, maintaining adequate patient ventilation is equally important regardless of the attempts made.” It is not only crucial for anesthesiologists. During an elective induction, there is always the (theoretical) possibility of reversing the anesthesia. This is not possible in an emergency setting as the indication for airway management is prevention of hypoxia and death.
- There is only one newer study (Zuercher, 2022, which included patients from January 2017 to July 2019!). How has the evolution of video laryngoscopy and the use of styles or bougies affected the relevance of an intubation LMA? Is this still used in routine practice? You could look for recommendations for difficult airway carts (e.g. https://www.ncbi.nlm.nih.gov/pmc/articles/PMC6369510/)

Conclusion:

- Please shorten your conclusion. The first two sentences can be omitted.
